# Cytologic Findings in Esophageal Perforation: An Institutional Experience With Pleural Fluid Specimens

**DOI:** 10.1002/dc.70111

**Published:** 2026-03-09

**Authors:** Mason Marshall, Samer Khader, Sigfred Lajara, Liron Pantanowitz

**Affiliations:** ^1^ Cytopathology Center of Excellence University of Pittsburgh Medical Center Pittsburgh Pennsylvania USA; ^2^ Department of Cytopathology Memorial Sloan Kettering Cancer Center New York New York USA

## Abstract

**Introduction:**

Esophageal perforation (EP) is a rare but life‐threatening condition, and most cases are due to iatrogenic causes. The rest occur spontaneously, due to malignancies, or trauma. The mortality rates can approach 50%, and delayed diagnosis of EP exacerbates patient outcomes. Cytologic recognition of pleural fluid specimens with oropharyngeal contents (e.g., squamous cells, *Candida* species) or food/foreign material can expedite the diagnosis of EP. This study presents our institution's experience with cytologic findings in EP patients.

**Materials and Methods:**

We reviewed our institutional database over a 25‐year period to identify pleural fluid cytology specimens from EP patients. Data on patient demographics, clinical history, and microbiology culture results were collected. Slides were assessed for squamous cells, food/foreign material, and infectious organisms.

**Results:**

Eleven pleural fluid cytology specimens from 10 patients with EP (seven males, three females; mean age 68 years) were retrieved. Three cases were atypical, and eight were negative for malignancy. Mortality occurred in four (40%) patients within a mean of 2.2 months post‐EP diagnosis. The majority of EP causes were iatrogenic (82%) or associated with malignancy (55%). Squamous cells were present in 27% of specimens, while food/foreign material and fungi were identified in 36% and 9% of cases, respectively. Cultures were positive in seven (64%) patients, with 46% showing bacterial growth and 55% positive for fungal organisms.

**Conclusions:**

Our experience corroborates that EP carries a high mortality risk, underscoring the need for prompt diagnosis. Although *Candida* species were present in only a small subset of pleural fluid samples, the presence of squamous cells or food/foreign material is a helpful diagnostic clue of EP, particularly in patients with a history of recent medical manipulation or malignancy involving the esophagus. Our findings highlight the potential of identifying involved pleural fluid cytology specimens to decrease the time to diagnosis of EP.

## 1 Introduction

Esophageal perforation (EP), defined by a full‐thickness breach in the esophageal wall, is an uncommon but life‐threatening condition. The incidence is estimated at 3 per 100,000 people, with an overall estimated mortality rate of 20% [1, 2, 3]. EP can pose significant clinical challenges to diagnose due to its wide range of clinical presentations and diverse etiologies [1, 2, 4]. This often results in delayed diagnosis, which worsens patient outcomes [1, 2, 4, 5, 6, 7, 8]. More than half of these cases arise iatrogenically as complications of medical procedures (e.g., upper gastrointestinal endoscopy), although spontaneous rupture, malignancy, caustic ingestion, or trauma are also known causes [1, 3, 4, 9]. Regardless of etiology, timely recognition and treatment is necessary to improve outcomes, underscoring the importance of early and accurate diagnosis.

Traditional diagnostic approaches for EP include imaging and esophageal endoscopy; however, cytologic examination of fluid specimens such as pleural fluid presents a potential rapid and easily accessible adjunctive diagnostic modality [5, 7]. Certain cytologic features, such as the presence of food particles, foreign material, or oropharyngeal contents (including squamous cells or *Candida* species), can serve as immediate indicators of EP [5, 7, 10]. Recognition of these elements in cytologic fluid preparations could expedite diagnosis, especially in cases of EP where imaging results are equivocal or invasive diagnostic procedures are contraindicated.

To further characterize the pertinent cytologic features associated with EP, we reviewed fluid cytology specimens collected from patients at our institution over the last 25 years. We describe the cytomorphologic findings in these samples associated with EP, correlate them with microbial culture results, and summarize the clinical profiles of affected patients. This study also aims to highlight the diagnostic value of potential cytologic indicators of EP and emphasize their potential role in facilitating early recognition of this high‐risk condition.

## 2 Materials and Methods

Institutional review board (IRB) approval was obtained. Thereafter, this retrospective study was conducted at our institution and included patients admitted between August 2000 and June 2025. We queried our institutional electronic pathology database (CoPath) to identify pertinent pleural fluid cytology specimens from patients with a confirmed diagnosis of EP. Inclusion criteria were: (1) a documented diagnosis of EP, (2) availability of at least one pleural fluid cytology specimen, and (3) sufficient clinical and demographic information for review. A total of 11 cytology specimens from 10 patients met these criteria and were included in the analysis. Demographic information, including patient age, gender, clinical history, and follow‐up information, were retrieved from the patients' electronic medical records. Patient outcomes were recorded, including survival status and, when applicable, time to death from initial presentation with EP.

Pleural fluids from these patients were collected and centrifuged at 2500 rpm for 5 min. Following centrifugation, supernatants were discarded, and the remaining cell pellets were used in preparation of both a Diff‐Quik stained Cytospin slide and a ThinPrep slide (Hologic, Bedford, MA) stained with a modified Papanicolaou protocol. Cell blocks were prepared on all specimens with sufficient material and were stained with hematoxylin and eosin. Cytology specimen results were reported using the standard cytology reporting categories: *Nondiagnostic, negative for malignant cells (NFM)*, *atypical cells present, suspicious for malignant cells (SFM)*, and *positive for malignant cells (PFM)*. In addition, all slides were retrieved and reviewed for the presence of squamous cells, food particles or foreign material, and infectious organisms (e.g., bacteria, fungi including yeast). When clinically indicated, microbiological cultures were obtained from fluid samples. Cultures were prepared and analyzed according to institutional laboratory protocols to identify bacterial, fungal, or other microbial pathogens.

## 3 Results

A total of 11 specimens from 10 patients with EP were identified for inclusion in the study (Table 1). There was a slight male predominance, with a M:F ratio of 2.3:1. Patients in our series were older adults with a mean age of 68 (range 41–83) years (Table 1). Specimens included 11 pleural fluids. Three cases were signed out as atypical (27%) and eight were NFM (73%). Most cases were attributed to iatrogenic causes (82%) or were associated with malignancy (55%). The five patients with a prior history of malignancy were comprised of two gastroesophageal junction (GEJ) adenocarcinomas, one esophageal adenocarcinoma, one pharyngeal squamous cell carcinoma (SCC), and one oropharyngeal SCC. Three patients had circumferential esophageal masses on recent prior endoscopic biopsies. Four patients (40%) died, with a median survival time of 2.2 months following their diagnosis of EP. Clinical presentations varied but commonly included chest pain, dysphagia, dyspnea, and hematemesis. A history of tobacco (73%) or alcohol abuse (45%) was present in all but one case (91%).

**TABLE 1:** Clinicopathologic and microscopic characteristics in patients with esophageal perforation.

Age	Gender	Cause	Clinical symptoms	Comorbidities	Category	Mortality?	Bacteria	Fungus	Food/debris	Squamous cells	Cultures
64	M	Iatrogenic‐ variceal banding and Blakemore tube	Hematemesis, jaundice, altered mental status	Alcohol abuse, cirrhosis, varices	Atypical	Yes (1 month)	Yes	No	No	No	*E. coli*, *Stenotrophomonas maltophila*
82	F	Malignant‐ esophageal adenocarcinoma. Iatrogenic‐ radiation and EGD with biopsy and dilation	Chest pain, dysphagia, dyspnea	Metastatic breast cancer, tobacco use, GERD	Atypical	Yes (1 month)	Yes	Yes	No	Yes, atypical	*Enterococcus faecallis*, *Lactobacillus* sp, *Candida glabrata*, *Candida albicans*
63^a^	M	Malignant‐ widely metastatic GEJ adenocarcinoma. Iatrogenic‐ EGD with dilation and stent placement	Chest pain, dysphagia	Tobacco use	NFM	Yes (6 months)	No	No	No	No	Yeast not cryoptococcus
63^a^	M	Malignant‐ widely metastatic GEJ adenocarcinoma. Iatrogenic‐ EGD with dilation and stent placement	Chest pain, dysphagia	Tobacco use	NFM	Yes (6 months)	No	No	No	No	X
83	M	Iatrogenic‐ TEE	Chest pain, dyspnea, fever	Dementia, GERD	NFM	Yes (1 month)	No	No	Yes	No	*Oropharyngeal flora*, *Saccharomyces cerevisiae*
41	F	Emesis	Chest pain, dyspnea	Tobacco use, alcohol use, GERD, Asthma	NFM	No	Yes	No	Yes, food material	Yes	*Streptococcus mitans*, *Lactobacillus* sp, *Candida albicans*
48	F	Iatrogenic‐ Heller Myotomy	Chest pain, dysphagia, dyspnea, nausea	Tobacco use, alcohol abuse, cirrhosis, achalasia, GERD, hiatal hernia	NFM	No	Yes	No	No	Yes	*Streptococcus angingosus*, *Candida dubliniensis*
64	M	Malignant‐ oropharyngeal squamous cell carcinoma. Iatrogenic‐ radiation, EGD for food impaction	Chest pain, dysphagia, dyspnea	Pharyngeal SCC, tobacco use, GERD	Atypical	No	No	No	No	No	No growth
77	M	Iatrogenic‐ EGD for food impaction. Emesis	Chest pian, hematemsis	Tobacco use, GERD	NFM	No	No	No	Yes, food material	No	No growth
83	M	Emesis	Chest pain, fever, somnolence	Alcohol use, congestive heart failure, GERD, hiatal hernia.	NFM	No	No	No	Yes, striated muscle fibers	No	*Candida albicans*
74	M	Malignant‐ laryngeal squamous cell carcinoma. Iatrogenic‐ laryngectomy, chemoradiation, rigid esophagoscopy and biopsy	Chest pain, dyspnea	Recurrent oropharyngeal SCC status post radiation and laryngectomy, GERD, COPD. Alcohol and tobacco use	NFM	No	No	No	No	No	No growth

^a^ Denotes same patient.

**FIGURE 1:**
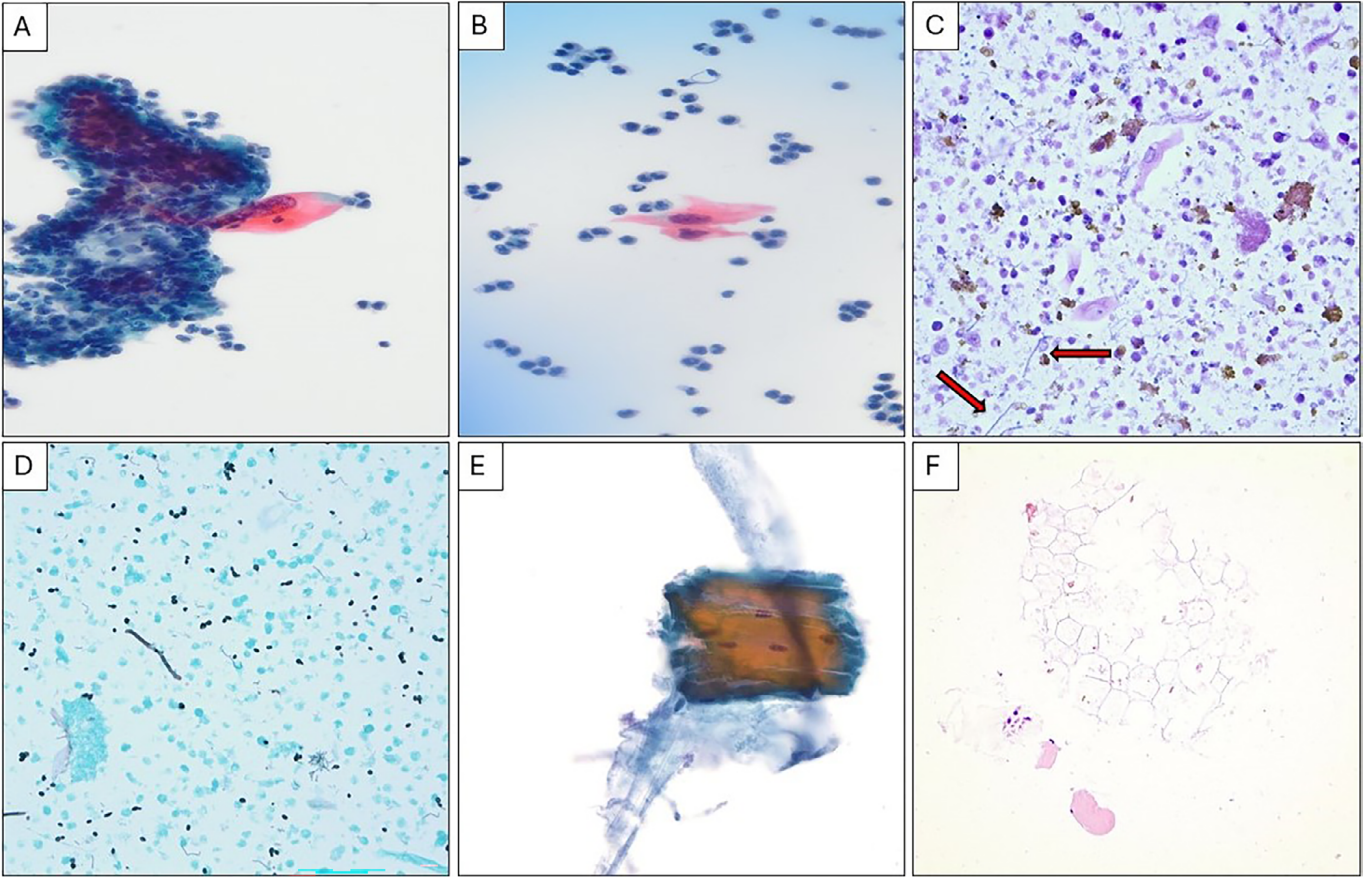
(A and B) Highly atypical squamous cells associated with acute inflammatory cells (Pap, 400×). (C) Squamous cells with abundant bacteria, fungal forms (arrows), hemosiderin, and extracellular debris (DQ, 400×). (D) Grocott's methenamine silver stain is positive for *Candida* species (GMS, 400×). (E) Striated muscle, indicative of ingested food material (E: Pap, 400×). (F) Degenerated vegetable matter, indicative of ingested food material (F: H&E, 200×). [Color figure can be viewed at wileyonlinelibrary.com]

Cytomorphologic findings included squamous cells in 27% of fluid specimens, particularly in cases associated with esophageal instrumentation or malignancy, suggesting a potential diagnostic clue. Notably, atypical squamous cells were present in one specimen (9%), from a patient with a history of malignancy (Figure 1A,B). Food particles or striated muscle fibers were observed in 36% of cases (Figure 1E,F), consistent with contamination from ingested material. Fungi were less commonly seen, with only one instance of *Candida* species identified on Grocott's Methenamine Silver (GMS) stain (Figure 1D), despite positive fungal cultures in five additional cases (55%). Overall, microbiological culture studies were positive in 70% of the specimens that were submitted. *Candida* species were cultured in 36% of cases, with two additional cases containing other fungal species. Bacterial growth was present in 45% of cases, with organisms including *Streptococcus* species, *Enterococcus faecalis* , *Stenotrophomonas maltophilia*, and *Escherichia coli* (Table 1).

## 4 Discussion

EP is rare, and therefore infrequently encountered by pathologists. In our 25‐year review at a large academic referral center, only 11 cases were identified. While it can occur in any age group, EP is most commonly reported in older adults, typically over the age of 60 years, with a male predominance, which are in keeping with the patient demographics reported in our series [1, 3, 9]. There were no pediatric cases in our series. Perforations can occur in any of the three esophageal compartments—cervical, thoracic, or abdominal, but thoracic perforations are by far the most common, comprising approximately 70% of the total cases, with cervical and abdominal each occurring in roughly 15% of cases [1, 3, 9, 11]. Given that the upper esophagus is reinforced by striated muscle, perforations (especially spontaneous) most commonly occur in the distal lower left esophagus. This area is vulnerable due to a combination of factors, including lack of supporting tissue, a rich neurovascular supply, and the junction of muscle fiber types which together create weak points [4, 8]. This consequently results in a left‐sided pleural effusion in 70% of cases when present [5, 7, 8]. Our data further supports these findings, as 66% of the pleural fluids that had laterality specified were left sided. The absence of a serosal layer makes the esophagus further vulnerable to perforations and limits containment once a breach occurs [1, 2, 12].

Perforation of the esophagus can be caused by a wide array of etiologies including iatrogenic, spontaneous (Boerhaave's syndrome), malignancy, foreign body ingestion, caustic injury, and trauma [1, 2, 4, 9]. Iatrogenic causes are the most common etiology, accounting for over half of cases [1, 2, 3, 4, 8, 9]. Our results are in line with the above findings, as 82% of patients had a history of recent esophageal manipulation. Examples of preceding procedures included esophagogastroduodenoscopy (EGD), frequently in combination with dilation or biopsy, Heller myotomy, variceal banding, and transesophageal echocardiogram. Spontaneous rupture represents the other significant etiology, occurring in 38% of patients in a recent large systematic review [9]. In a previously reported series of 142 patients, approximately 7% developed EP secondary to malignancy [11]. The findings in our small series were slightly less, as no patients had a malignant perforation. However, 55% of the cases in our series did have a history of esophageal or oropharyngeal malignancy (three esophageal/GEJ adenocarcinomas, one laryngeal carcinoma, and one pharyngeal carcinoma). Interestingly, all but one patient in our series had either a history of tobacco (73%) or alcohol abuse (45%) (Table 1). In addition to being known carcinogens, these substances can exacerbate pre‐existing esophageal diseases and further weaken the esophageal wall [4]. Alcohol‐induced emesis is also a known trigger of “spontaneous” rupture or Boerhaave's syndrome [1, 4]. Three patients in our series (27%) presented with emesis. It is postulated that emesis induces a forceful and sudden increase in the intraluminal esophageal pressure that leads to subsequent perforation. Furthermore, eight patients (80%) had underlying gastroesophageal reflux disease (GERD), a known risk factor for EP development [4, 13]. In addition to the direct damage of the esophageal mucosa by refluxed stomach acid, patients with GERD frequently undergo surveillance EGD with biopsy to monitor progression to Barrett's esophagus, further increasing their risk of EP development.

EP can be challenging to diagnose clinically and is frequently missed. In one large series, approximately 20% of EP cases were identified only at autopsy [3]. Several factors contribute to this diagnostic challenge. First, the clinical symptoms and presentation are nonspecific, and they vary depending on the anatomic location of the perforation [1, 4]. In addition, the classical Mackler's triad (vomiting, chest pain, and subcutaneous emphysema) is only present in approximately 25% of cases [2]. Imaging studies may be negative if taken shortly after onset of the perforation, or if the leak is self‐contained, while a subset of perforations are not detected endoscopically [1, 2, 5, 7]. Lastly, the rarity of the condition also limits physician experience, potentially hindering recognition [2]. These difficulties all contribute to delayed patient diagnosis and therefore poorer prognosis. Approximately 40% of patients are admitted to the hospital greater than 24 h after perforation; the same review indicated that sepsis was present upon admission in one‐quarter of cases [9]. Early diagnosis is critical, as delays are associated with more invasive surgical therapy and higher patient mortality [2, 4, 8].

Breach of the esophageal wall may result in leakage of gastric, salivary, and biliary contents which cause chemical mediastinitis and typically a left‐sided pleural effusion [1, 8]. Within hours, a polymicrobial infection develops which can result in sepsis and septic shock [1, 2]. The poor prognosis observed in our cohort, with a mortality rate of 40% and median survival of only 2.2 months, underscores the severity of EP and the critical importance of rapid diagnosis and intervention. In two large series, the overall mortality rates of patients with EP were 13% and 17% [3, 9]. However, mortality rates are estimated to approach 60% when the diagnosis is delayed for greater than 24 h [8]. While our cohort demonstrated a worse overall prognosis compared to the literature, 50% of our patients had a history of malignancy which has been demonstrated to be an especially poor prognostic factor [14]. Given that nine of the cases had an iatrogenic etiology and six had a history of malignancy, our findings also emphasize the need for heightened vigilance in patients with recent esophageal manipulation or underlying malignancy, as these factors predispose individuals to EP.

The diagnosis of EP traditionally centers around a combination of clinical evaluations and imaging or endoscopic findings [1, 2, 4]. A plain chest radiograph is often ordered initially to assess for pneumothorax, pleural effusion, and pneumomediastinum. Contrast esophagography remains the gold standard, with extravasation of swallowed contrast confirming the diagnosis. Endoscopy in the setting of acute perforation is controversial, but is generally reserved for cases with negative or equivocal imaging [2, 4]. Our study highlights the potential utility of pleural fluid cytology as a rapid and accessible diagnostic adjunct in EP. Cytologic evaluation can be especially useful in cases where imaging findings are equivocal or when contrast or endoscopy is contraindicated. Most cytology laboratories should be able to complete pleural fluid cytologic processing within a few hours of specimen collection, allowing for potential rapid diagnosis or confirmation of EP. We analyzed 11 fluid cytology specimens from 10 patients with EP in our institution, finding that certain cytologic features—particularly the presence of squamous cells, food particles or foreign material, and microorganisms—can provide valuable diagnostic clues of EP. Despite the relatively limited number of cases in our series, these findings further contribute to the growing evidence that fluid cytology can be a useful adjunct in the challenging and urgent setting of EP [5, 7, 10]. In addition, the fluid collected can be sent for biochemical analysis and microbiological cultures that can further assist or support in the diagnosis of EP [5, 6, 8, 10]. The fluid of EP is typically transudative and displays non‐specific markers of inflammation and infection, such as elevated lactate dehydrogenase (LDH), decreased glucose from microorganism consumption, and an elevated white blood cell count rich in neutrophils. More specifically, the fluid should have a low pH and high amylase due to the leakage of gastric contents and salivary fluid, respectively [6]. However, it is important to note that while elevated pleural fluid amylase levels can be suggestive of EP, they can also be seen in pancreatitis, tuberculosis, empyema, rheumatologic or autoimmune conditions, and even malignancy [6, 7, 10, 15].

The presence of squamous cells in approximately one‐quarter (27%) of our cases suggests that this finding may serve as a useful diagnostic clue, particularly in patients with recent esophageal instrumentation or a history of malignancy (Figure 1A,B). Squamous cells in fluid cytology can be indicative of esophageal disruption, such as a fistula if chronic, and previous studies have similarly reported that their presence in pleural fluid can indicate EP or other esophageal pathology. The squamous cells in our series were atypical in one and benign with reactive changes in two cases. This study confirms that while squamous cells alone are not definitive for EP, their presence in conjunction with relevant clinical history and imaging can strongly suggest a diagnosis of EP, especially in settings where confirmatory diagnostic tools may be limited or delayed. The differential diagnosis for atypical squamous cells in pleural fluids, aside from esophageal SCC, includes SCC of the lung, metastatic cervical, cutaneous, laryngeal, or oropharyngeal SCC, and ruptured mediastinal teratoma [5]. Benign squamous cells may be seen with skin contamination that occurs with a pleural tap. The cytologic features of esophageal SCC are similar to other SCCs in fluid cytology; viz., pleomorphic shaped “tadpole” cells with dense orangeophilic keratinizing cytoplasm and markedly irregular, pleomorphic, and hyperchromatic nuclei [14]. Importantly, as iatrogenic injury is the leading etiology of EP, caution is advised as specimens taken shortly after manipulation have been demonstrated to contain markedly atypical mesothelial cells which can be misinterpreted as adenocarcinoma [14].

Food particles or foreign material and fungi were present in only a minority of cases (36% and 9%) (Table 1), indicating that these features may be less sensitive indicators of EP than previously thought [5, 7, 10]. It has been suggested that high‐speed centrifugation concentrates food and foreign debris, which may enhance detection [7]. Prior reports also indicate that food particles or debris may be grossly visible in some samples, facilitating a more rapid diagnosis [5]. In our cohort, fungal organisms were microscopically detected in only one specimen, though six cases (55%) were culture‐positive (Figure 1C,D). Apart from sampling bias, the low rate of identifiable fungal and foreign material may be due to the relatively advanced state of infection and degradation by the time of specimen collection, brisk obscuring inflammation, or low numbers of infectious organisms. When present, *Candida* species are highly suggestive of a gastroesophageal source of contamination. In one large series of pleural empyemas, *Candida* species were isolated in all cases caused by gastrointestinal perforation and absent from all other etiologies [10]. Others have suggested that *Lactobacillus* species, identified in cultures of two specimens, are also indicative of gastroesophageal contamination [6]. Our results suggest that while the presence of food particles and *Candida* species can be diagnostic, their absence does not rule out EP and should not delay further workup. Cytologic examination, paired with culture data, may thus play a key role in identifying infectious organisms early, allowing for more targeted antimicrobial therapy that could potentially mitigate morbidity and mortality.

This study has several limitations. First, it is retrospective and includes a small sample size, which may restrict the generalizability of our findings. Additionally, cytologic examination was only conducted on pleural fluid specimens, which may limit the applicability of our findings to other body fluids. We also had no cases with malignant squamous cells and few with fungal organisms or food particles microscopically.

In conclusion, our findings suggest that while cytologic examination of pleural fluid specimens may not replace traditional diagnostic modalities, it can play a valuable adjunctive role in rendering a timely diagnosis of EP.

## Author Contributions

Data analyses, manuscript draft, table: Mason Marshall. Conceptualization, figures and manuscript editing: Samer Khader and Liron Pantanowitz. Manuscript editing: Sigfred Lajara.

## Funding

The authors have nothing to report.

## Conflicts of Interest

The authors declare no conflicts of interest.

## Data Availability

All data analyzed during this study are included in this published article. All patient information has been de‐identified in accordance with institutional and ethical guidelines. The data are not publicly available due to privacy restrictions.
